# Supplementary material for: Are carbon emissions trading and green financial instruments synergistic? -Comprehensive quantitative research based on content analysis
Source: PLoS One. 2024 Mar 7;19(3):e0298601. doi: 10.1371/journal.pone.0298601 (PMC10919601; doi:10.1371/journal.pone.0298601)
Supplement: S1 Data — (ZIP) [file pone.0298601.s001.zip › Data/地级市Z_F工作报告.zip!/┴╔─■/╡ñ╢1⁄2╩╨/└┤╫╘╣1⁄2╓┌║┼í╛┬φ┐╦╩2╛▌═°í┐_2014.docx]

各位代表：

　　我代表丹东市人民政府向大会报告工作，请予审议，并请市政协委员和各位列席人员提出意见。

　　一、2013年主要工作回顾

　　一年来，在丹东市委的正确领导下，在市人大、市政协的有力监督和大力支持下，市政府带领全市人民，积极应对严峻复杂的经济形势，负重前行，迎难而进，较好地完成了年初确定的各项目标任务。

　　(一)国民经济主要指标平稳增长。预计全市地区生产总值完成1117亿元，比上年增长12%;规模以上工业增加值420亿元，增长13%;全社会固定资产投资1050亿元，增长20%;社会消费品零售总额432亿元，增长15%;外贸出口总额31.6亿美元，增长10%;实际利用外商直接投资10亿美元;引进内资项目到位资金498亿元，增长20%;公共财政预算收入实际完成136亿元，增长6.5%;预计城镇居民人均可支配收入21588元，增长10%;农民人均纯收入12685元，增长11%。

　　(二)项目落地和实施卓有成效。去年初，经过反复论证，确定了150个亿元以上新开工项目，通过采取与县(市)区、经济区签订责任状和项目包保等措施，促进了项目落地，目前中朝新鸭绿江公路大桥口岸物流区，丹东港大东港区、海洋红港区疏港高速，东北亚国际商贸城，三义高端氧化钙，参仙源酒业等年初确定的大项目均已开工建设。东北东部铁路客货运全面通车。三湾水利枢纽工程落闸蓄水。努力解决动迁难题，确保了沈丹客专和丹大快速铁路按进度施工，路基、桥涵建设进入收尾阶段。新鸭绿江公路大桥实现主塔封顶、主桥合龙。大东港区20万吨级矿石泊位进入设备安装阶段。大宇海洋重工、史密斯菲尔德现代农业、生物基尼龙56纤维等一批重大项目落户丹东。东港临港物流园区、博恩高新精密产业园、宗裕水上大世界等项目正在积极推进。

　　(三)工业经济稳定运行。面对工业经济遇到的巨大压力和困难，积极采取市领导包扶重点企业、出台中小企业扶持政策等措施，帮助企业解决难题、开辟市场，促进了部分停产、半停产企业恢复生产，稳住了工业的基础地位。预计全市规模工业主营业务收入1360亿元，增长7%。新增亿元以上企业40户、规模以上企业32户。日林集团进入中国企业500强行列。中小企业公共服务平台建成并通过国家级示范平台考核，为186户中小企业担保贷款7.5亿元。重点产业集群主业收入增长22%，新增两个省级重点产业集群，百亿元产业集群数量达到3个。博斯亚糖业、黄海新能源汽车基地、五一八船用曲轴等重大工业项目进展顺利;帕斯特谷物二期、本钢丹东不锈钢冷轧板、禾丰成三饲料加工、佰山木制品等一批投资10亿元以上重大工业项目实现竣工，亿元以上重点项目竣工投产29个。全市高新技术企业突破50户，高新技术产业增加值增长20.7%，省级高新技术产业开发区通过专家评审。中科院丹东产业技术创新与育成中心正式启动，3个省级重点产业集群拥有了研发公共服务平台。

　　(四)丹东新区、大孤山等区域呈现较快发展态势。丹东新区实现项目入驻和功能完善齐头并进，仪器仪表产业基地新签约入驻企业26户，钟表园、视光园建设正式启动，恒和国际贸易中心、国际会展城、滨江凯旋门等重大项目开工建设。新区医院加快推进，鹰桥国际学校招生开学，唐宁壹号商业街、科技五金城等商业综合体投入使用，近200户各类便民商业网点对外营业，新增的221路、303路公交车上线运营。在新区举办了全国十二运火炬传递、女篮比赛、房交会等大型活动，特别是第二届中朝经贸旅游文化博览会吸引了国内外参展、参会客商和群众约25万人次。大孤山经济区初具形象，基础设施建设整体铺开，“三纵两横”路网全线启动，供水、供电等项目加快推进，精密加工产业园区11个规模以上工业项目实现入驻，渤海石化等重大项目正在积极推进。翡翠湾开发全面实施，23平方公里区域规划编制完成，香港佳兆业房地产开工建设并实现销售，新东方商贸城项目已经启动，与香港南大洋、南阳豪盛、大连裕景等企业达成投资意向，浪头港区游船码头及配套区项目进入方案设计阶段。

　　(五)对外开放工作成绩显著。实际利用外资连续3年突破10亿美元。外贸出口实现逆势稳定增长，出口企业规模和质量不断提升，企业总数近1000户，其中出口超亿美元企业3户，超5000万美元企业10户，东港水产品等3个出口基地被确定为省级出口基地。对朝贸易继续巩固，中朝博览会期间，朝鲜参展商社108家，现场成交额1.35亿元，达成投资、贸易合作意向93项，意向金额16亿美元。对外经济技术合作健康发展，技师学院被认定为我市首家省级外派劳务培训基地。积极开展招商引资工作，全年“走出去”109次，“请进来”159次，邀请客商1700多人，香港、上海、深圳等地专题招商活动取得了预期效果。丹东港预计完成货物吞吐量1.2亿吨，集装箱吞吐量150万标箱，迈入亿吨大港行列，在东北三省和内蒙古等地建成陆港21个，到长春、牡丹江等8条海铁联运专线先后启动，东北东部区域合作进一步推进。丹东沿边开发开放试验区报批工作取得实质性进展。

　　(六)农业和县域经济取得新成果。全年粮食产量达到95.8万吨，连续三年获得大丰收。水产品产量60.4万吨，海洋与渔业经济稳居全省第二位。主要粮食作物耕种收机械化率达到72%。首个面积1万平方米的蔬菜育苗中心成立。在全国率先开通了“12316金农热线”服务平台。县域重点工业园区和产业集群实现规模和效益同步增长。全市5个10亿元以上农产品加工项目中的3个实现竣工投产，2个部分竣工投产。新增12个亿元以上农产品加工项目，规模以上农业产业化龙头企业达到200户。创建省级以上农产品出口示范区4个，农产品出口额增长10%。“一县一业”产业水平逐步提高，东港出口草莓、宽甸出口板栗质量安全示范区被批准为国家级示范区;凤城市被命名为“中国板栗产业示范市”，成为全国首个获此殊荣的县(市);宽甸石柱参入选首批中国重要农业文化遗产名录。“青山工程”完成治理面积7775亩，“两退一围”面积3万亩，大梨树、河口、獐岛、五龙背4个村(镇)被授予“中国美丽乡村”称号。农建水利工作进一步加强，我市成为“全国水生态文明”试点城市。采取有效措施，成功应对9次强降水过程，全地区实现了安全度汛。动物疫情得到有效防控。

　　(七)城区经济和服务业快速发展。陆续向城区下放管理权23项，城区发展潜力得到进一步激活。万达广场正式投入运营，鹏欣水游城地下商业工程正在施工，华美新天地开工建设，兴隆大家庭落户宽甸，重大城市综合体项目正在逐步带动形成新的商圈。希尔顿、博恩御山水等5个五星级酒店加快建设。开展主题促销，推动“夜经济”繁荣，保持了消费市场持续活跃。旅游业发展态势良好，宽甸成为全省首个生态旅游实验区，温泉、沟域、乡村、江海、边境旅游持续火爆，北黄海温泉小镇等4个投资超亿元的温泉旅游度假项目开工建设;旅游宣传力度进一步加大，盛夏“凉爽丹东”成为城市新名片;国家级赴朝旅游集聚区项目已申报至国家旅游局。认真落实调控政策，房地产业保持健康发展态势。

　　(八)财政金融实现增长。公共财政预算收入中的税收收入突破100亿元，增幅高于公共财政预算收入增长，税收结构不断优化，收入质量稳步提高。公共财政预算支出突破200亿元，支出重点继续向民生等领域倾斜，支出增量的80%以上用于保障和改善民生。预计全市本外币存款余额1350亿元，增长12.5%;贷款余额785亿元，增长14.8%;存贷比进一步提高，达到58%。新增贷款连续4年突破百亿元，各类融资220亿元。辽阳银行、阳光财险和金元证券等金融机构进驻我市。

　　(九)民生工作较好落实。市政府承诺办好的菜篮子、城乡饮水安全、百姓健康、群众温暖、老旧小区改造、畅通工程、就业和社保体系完善、特殊困难群众关爱八大民生工程全面展开。老城区改造力度加大，大修道路30余条，人民街、金海路、福春街完成了拓宽改造，四号干线锦江山隧道贯通工程已完成招投标，正在开展周边动迁工作。振八街等5个铁路道口“卡脖”问题得到解决。通过滨江中路地下停车场建设及站前广场、女人街周边等区域改造，新增停车位约2000个。竣工保障性住房7000余套，实施棚户区改造20余万平方米，全市最大的工矿棚户区五龙金矿棚户区22栋9万平方米安置房已全部封顶。东升路、解放后路等排水工程的建设、改造，缓解了城市东部地区低洼积水行路难状况。新增4万城市居民和11万农村人口喝上了安全饮用水。加大环境生态保护工程投入，实施“蓝天工程”，集中供热联网140万平方米，完成既有建筑供热计量节能改造53万平方米，城区空气质量优良率达到97.8%。城市绿化和内河整治有序开展。金山、五龙背、青城子等乡镇污水处理厂主体工程完工。“四城联创”工作取得新进展，已经具备国家卫生城和环保模范城申报资格，全省文明程度指数测评取得第三名。实施乡镇环境卫生综合整治，较好地解决了农村脏乱差问题;开展清理私搭乱建专项行动，拆除城区各类违章建筑7000余处，城乡环境有了极大改观。全年实现实名制就业6万人，城镇登记失业率控制在4.2%以内。社会保险扩面征缴年度任务全面完成，社保待遇水平稳步提升。城市低保标准由每人每月365元提高到410元，农村低保由每人每年2180元提高到2580元，农村五保供养和各类优抚对象生活补贴标准大幅提高。资助近10万低保对象参加城镇居民医疗保险和新农合。建成1处城市、13处村镇公益性公墓。圆满完成全运会丹东赛区各项工作，举办马拉松赛等各项群众体育活动，在全市范围内投放群众健身器材100余套，万隆体育场整修一新。基础教育强县(市)区扎实推进，职业教育不断加强，改扩建20所乡镇以上公办幼儿园，完成114所义务教育标准化学校建设，教育公平得以促进，阳光分班全面实施。公共文化服务体系建设积极推进，工作成果被文化部评为国家示范项目。非政府办基层医疗机构基本药物制度和公立医院改革稳步推进。在全市范围内实施了免费孕前优生健康检查。食品药品安全保障水平不断提高。

　　(十)民主法制和社会治理工作有了新进步。自觉接受市人大及其常委会依法监督和政协民主监督，积极听取各民主党派、工商联、无党派人士和人民团体意见。

　　办理市人大代表建议、政协提案726件，办结率和满意率均达到100%。精神文明创建活动扎实推进，以志愿服务为主要内容的“文明使者在行动”广泛开展。民兵预备役和双拥工作取得新成果。民族工作和依法管理宗教事务水平进一步提高。依法行政扎实推进，“六五”普法有效实施，为全市困难群体提供法律援助8200件次。行政审批制度改革不断深化。政务公开稳步向基层延伸。顺利完成村委会换届选举工作。审计监督职能得到发挥。农村集体资金、资产、资源委托代理和网上监管全面铺开，“五大系统”建设深入推进，严厉查处了损害群众利益的违法违纪问题。认真做好信访积案化解，妥善解决了大量信访诉求。社会管理综合治理得到加强，应急管理体系不断完善，安全生产形势总体平稳，平安丹东建设成效明显，边海防管控水平有效提升，国防动员工作取得突破，保持了边境地区和谐稳定。

　　外事、侨务、台务、口岸、统计、物价、保密、档案、接待、地方志、人防、气象、防震减灾、妇女儿童、老龄、残疾人等工作也取得了新成绩。这些成绩的取得，是省委、省政府和市委正确领导的结果，是市人大、市政协大力支持的结果，也是全市人民共同努力的结果。在此，我代表市政府向全市各族人民，向各民主党派、各人民团体和其他各界人士，向驻丹中省直单位和驻丹部队、武警官兵表示衷心的感谢并致以崇高的敬意!

　　各位代表，在肯定成绩的同时，我们也清醒地认识到，全市经济社会发展还存在一些亟待解决的问题，突出表现在：一是经济下行压力依然很大，主要经济指标增速放缓，经济结构调整不快;二是重大项目尤其是工业大项目引进不多，推进较慢，受大环境影响，一些竣工项目迟迟不能投产、达产;三是企业自主创新动力不足，低端产品占比高，市场竞争力和抗风险能力较弱;四是公共财政收入增长放缓，民生等刚性支出明显加大，政府债务进入偿还高峰期，财政运行更加困难;五是政府系统工作作风虽有改进，但官僚作风、浮夸风还不同程度地存在，服务意识和水平有待进一步提高。这些问题已引起我们的高度重视，在今后的工作中，将采取切实有效的措施加以解决。

　　二、2014年经济社会发展主要任务和重点工作

　　2014年，是深入贯彻落实党的十八届三中全会精神，全面深化改革、扩大开放的重要一年，我们将按照中央“稳中求进”的工作总基调，抢抓机遇，奋力前行，把丹东的各项工作真正落到实处。为此，我们确定今年丹东经济社会发展的总体思路是：认真贯彻落实党的十八届三中全会精神，按照市委十一届七次全会的要求，把改革贯穿于全年各项工作，坚定不移地扩大开放，全力抓好三次产业稳步增长和结构优化调整，重点实施新区、大孤山、翡翠湾、临港区域、高新区的开发建设，加快推进老城区改造提升，使新老城区形成各有特色的增长极，促进经济高质量持续健康发展、社会和谐稳定，促进发展成果更多更公平惠及全市人民，为率先全面建成小康社会奠定基础，努力把丹东建设成为“文明、生态、繁荣、富裕”的宜居佳地。

　　2014年，全市经济和社会发展的主要目标是：全市地区生产总值比上年增长10%，公共财政预算收入增长10%，全社会固定资产投资增长18%，城镇居民人均可支配收入增长10%，农民人均纯收入增长9%，社会消费品零售总额增长12%。

　　为此，今年将全力抓好以下重点工作：

　　(一)加快实现沿江沿海“五点布局”率先崛起

　　进一步促进新区繁荣。加快新区功能完善和项目推进，完善医疗、教育、交通、市场等配套设施，完成辽东学院迁建项目选址等工作，实现实验小学新区分校挂牌招生，新区医院整体建成，仁和商业街、国门湾国际商贸城、新加坡城商业街、牛顿金园大厦等商贸项目开业运营，继续增加新区交通公交线路和车辆。推进纳诺工业城、国际会展城、手表珠宝园、世贸中心等项目实施。

　　全力抓好大孤山经济区建设。加快临港产业园、精密加工产业园、“三纵两横”路网以及疏港铁路等基础设施建设。强化重化工业招商引资，推进海洋红港区、产业园区及城区联动发展。精密加工产业园区实现新入驻规模以上企业20户。力争丹东石化搬迁改造项目开工，木制品精深加工、高新石油装备制造等项目加快建设，海洋红港区一个5万吨级通用泊位竣工，百万吨造纸项目有重大进展。

　　不断加快翡翠湾建设步伐。实现区域内路网和机场至帽盔山城市景观路开工。启动游船码头及配套区建设。跟踪服务好佳兆业房地产等已开工项目，争取新东方国际商贸城、富地城、汤池汽车4S店集聚区和二手车交易市场等项目开工建设，完成丹东化纤公司、黄海汽车厂等土地收储，争取翡翠湾沿江一线、胜利街北侧落地一批高端商贸地产项目。

　　大力发展临港区域和产业。抓住北黄海开发机遇，实施“以港兴市”战略，确保大东港区20万吨级矿石泊位投入使用，加快散杂货、石油化工、集装箱等泊位建设。临港产业重点实施好现代农业综合项目和船舶重工项目，推进帕斯特谷物三期工程开工建设并实现当年竣工投产，大宇海洋重工项目建造船舶10艘以上，5万吨级船坞建成投入使用。加大港口对东港经济区的辐射，加强产业融合和对接，进一步拉长临港产业链条，抓好东港临港物流园区等项目建设，促进仓储、物流业的发展。

　　积极推进高新技术开发区建设。实现尼龙56项目一期开工，力争当年竣工投产，带动防护纺织制品产业集群提升规模。引导我市现有高新技术企业向高新区集聚，使其尽快成为我市工业发展新高地。同时，依托高新区带动智能煤气表、纳米印刷制版等高新技术产业发展，在全市范围内重点培育好30户科技型企业。加强与中科院、大连理工等科研院所的产学研合作，加快科技成果转化，提高企业自主创新能力。

　　(二)不断促进工业经济实力稳步壮大

　　做大做强骨干企业。深入开展“三百企业工程”，通过政策扶持和资源整合，鼓励曙光、五一八等一批骨干企业实现产业升级、投资合作以及跨地区兼并重组，引导更多企业建立和完善现代企业制度。力争新增亿元以上企业30户，10亿元以上企业2户，50亿元以上企业1户。

　　扶持提升中小企业。实施创新驱动战略，落实好中小企业“超亿、进规”奖励政策，抓好一批创新示范企业。发挥好“中小企业服务平台”和“产品推广与市场营销”等服务平台作用，帮助中小企业开拓市场、融资担保，力争新增规模以上企业40户。

　　加快发展重点产业集群。积极推进新能源汽车等重大工业项目建设，形成产业集群和园区的龙头。加快工业园区基础设施建设，加大专业招商力度，引导支持同类企业向园区集聚，延长上下游产业链条。建立和完善仪器仪表、防护纺织制品、汽车及零部件、再生资源等重点产业集群公共服务平台。

　　切实提高工业经济运行质量。通过市领导包扶企业、实施重点企业联络员制度、强化监测分析等措施，及时掌握企业运行状况。发挥生产要素协调服务机制作用，帮助企业解决难题，促进有潜力的停产、半停产企业恢复生产。

　　(三)继续保持县域和农村经济较快增长

　　加快县域经济发展。抓好县域重点工业园区和产业集群建设，努力培育超百亿元产业集群。利用政策扶持，做大做强“一县一业”。继续抓好精品农业项目和相关龙头企业建设，实现在建的10亿元以上农产品加工项目全部竣工投产，亿元以上项目加快推进，规模以上农业产业化龙头企业达到220户。

　　提升现代农业比重。加快“兴边富民”特色优势产业建设，实施种植业生产结构调整。继续抓好“菜篮子”工程，引导、扶持城市周边农民发展蔬菜、食用菌、小浆果等优势特色产业，扩大无公害蔬菜生产规模。以粮食高产、精品果蔬、现代渔业发展为重点，打造一批名特优农产品示范基地，做大做强东港大米等农产品品牌。大力推广生态养殖模式，加快病死动物无害化处理。加强农业科技创新，做好“阳光工程”和设施农业培训。构建新型农业经营体系，规范农民专业合作社，鼓励、扶植家庭农场发展。农产品出口创汇增长10%。继续提高农业机械化水平。力争海洋红中心渔港移地换建和前阳一级渔港建设取得实质性进展。

　　稳步推进城镇化建设。深入开展城乡环境卫生综合整治，将整治工作和基本公共服务向全地区所有行政村延伸，加大农村基础设施建设力度，切实改善农村生产生活条件，建设好美丽乡村。逐步建立城乡统一的建设用地市场，加快乡镇规划修编，继续实施移民康居工程，引导农村人口向县、镇集聚。加快兴隆大家庭、林荫国际等县域城市综合体项目建设。

　　(四)着力提升服务业发展水平

　　进一步活跃城区经济。壮大经济规模，突出城区服务业的引领作用。完善服务业集聚区发展规划，提高新兴业态服务业集聚区的比重，抓好天赐国际金融中心等新兴业态服务业项目。新增1个省级服务业集聚区，使总量达到4个。改造和提升老商圈。以万达广场为核心，整合新乐购、锦绣家居、安东老街、鹏欣水游城等商业业态，培育和打造新商圈。力争在建的五星级酒店在两年内全部建成投入使用。发展好“夜经济”，引导餐饮业向特色化、大众化、健康化方向经营。继续保持房地产业健康发展。

　　大力发展特色旅游。加强旅游基础设施建设，提升旅游文化内涵，打造具有包容性的城市思想和文化特质，努力实现我市旅游全域化、全年化、全民化。做好辽宁(宽甸)生态旅游实验区总体规划编制、重点项目策划、优质旅游产品打造以及扶持政策争取。加快推进鸭绿江精品旅游带建设，抓好鸭绿江、天桥沟5A级旅游景区及大梨树、青山沟、黄椅山、大鹿岛4A级景区创建。提升温泉、江海等传统旅游项目，开发湿地、海角、历史文化遗迹等新特旅游项目，支持和鼓励农家乐扩大规模、提升档次。加强旅游宣传和促销，全力策划、举办好观鸟节、桃花节、枫叶节、采摘节，以及中韩中小企业旅游商品博览会。突出赴朝旅游特色，积极争取两日游、边境登岸游、登岛游等新项目，力争国家级赴朝旅游集聚区早日获批。

　　(五)不断加快对外开放步伐

　　强化招商和项目储备。依托我市资源优势和产业基础，紧盯世界500强、国内行业500强企业，积极组织开展对日韩、香港、台湾，以及珠三角、长三角、东北等国家和地区的招商活动。精心筛选、谋划、储备一批符合国家产业政策的优质项目。

　　狠抓项目落地和竣工。对亿元以上重点开工项目，继续实行县(市)区、经济区目标责任制，明确责任人、时间进度和保障措施，全年新开工超亿元项目100个以上。积极破解丹大铁路、沈丹客专等项目动迁难题，加快项目立项、环评、规划、用地等环节审批速度，对已开工项目要做好跟踪推进工作，及时解决出现的问题，确保项目按时竣工、投产、达产。

　　做好外资和外贸工作。加快外资项目的引进，扶持发展重点外资企业，推进丹东港境外上市融资，确保实现利用外资再次突破10亿美元。外贸出口在巩固朝、日、韩等传统市场的同时，积极开拓欧盟、非洲、南美等新兴市场。力争打造汽车零部件、草莓省级出口基地。

　　促进开放基础条件提升。加速各类口岸建设、升级和开放，新鸭绿江公路大桥口岸综合服务区和通关区达到通关条件。加强与国家有关部委衔接沟通，尽快实现边境经济合作区区位调整，积极争取国家在口岸监管、土地利用、劳动用工等方面的政策支持。力争丹东沿边开发开放试验区尽早获批，《东北东部经济带发展规划》早日列入东北振兴新十年规划。

　　(六)切实提高财政金融保障能力

　　保持财政平稳运行。进一步完善收入征管体系，努力培植新兴财源，切实提高收入质量。积极争取上级补助，提高财政保障能力。扎实推进预算绩效管理，调整和优化财政支出结构，持续加大民生等重点领域投入力度。防范和化解财政金融风险，严格政府债务发行、使用和管理。从严控制一般性支出，降低行政成本，提高财政资金使用效益。

　　实现多渠道融资。提高直接融资比重，引导民间资本进入金融行业，力争全年实现各类融资240亿元，新增上市公司1家、“新三板”挂牌企业3家、银行机构2家、担保机构3家。

　　(七)全力做好民生工作

　　道路施工改造方面。继续实施道路畅通工程，缓解城市交通拥堵压力。大修道路14条，实施四号干线锦江山隧道贯通工程和东楼线、黄海路、花园东路建设、改造，完善二号干线配套工程，完成国桢路铁路隧道拓宽工程和兴一路铁路道口改造。加大“公交先行”建设力度，增加公交车投放量，配套建设公交线路停车场、港湾车站。新增公共停车位2000个。启动民主桥、振安桥等桥梁安全检测和维修加固工程。

　　百姓安居方面。加大城市棚户区、工矿棚户区、城中村、老区危房改造力度，切实解决产权办理、小区物业管理、住宅修缮等涉及群众安居的切身利益问题。继续推进集中供热联网工程，实施既有住宅供热计量和节能改造。实现全市老旧小区基础数据建档和规范管理。继续实施城市燃气等地下管网、住宅小区自来水二次加压泵站改造;实现三湾水利工程四道沟水厂竣工，市水质监测中心开工建设，确保城市市政公用设施安全运行。

　　公园广场改造方面。对锦江山、元宝山、帽盔山等公园进行维修改造，新建珍珠山公园，实施新区安民山、灯塔山公园及周边开发。扩大合作区文化广场面积，将其建造成为我市首个音乐广场。

　　社会保障和就业方面。实施激励政策，引导被征地农民、外来经商务工人员实现养老保险应保尽保。实行基本医疗保险市级统筹。切实提高农民养老保险和医疗保障水平。全年培训下岗失业人员2.1万人，实现实名制就业6万人，年度高校毕业生就业安置率97%以上，城镇登记失业率控制在4.5%以内，零就业家庭保持动态为零。

　　扶贫帮困方面。积极做好135个贫困村脱贫帮扶工作。新解决6万以上农村人口饮水困难问题。免费为全市低保和低保边缘户残疾人办理人身意外伤害保险。为困难群体提供法律援助1万件次。建立申请低保家庭经济状况核对机制。积极推进城乡公益性公墓建设。

　　生态建设方面。深入开展“四城联创”，依法管理城市，巩固私搭乱建整治成果，抓好难点攻坚，严厉查处动迁过程中抢建抢栽等骗取动迁资金的违法犯罪行为。做好花园河等城市内河治理和城区旱厕改造。加强城乡生态环境建设，大力实施蓝天、碧水、青山工程，推进节能减排，淘汰落后产能。继续开展打击非法采挖移植山林树木行动，鼓励发展适合农村的新能源项目，加快生态市创建工作，使丹东拥有全省最佳的生态环境。

　　(八)全面推进社会事业发展

　　加强文化教育卫生体育等工作。落实教育领域综合改革任务，继续增加公办幼儿园数量，抓好市中小学素质教育基地和义务教育标准化学校建设，推进普通高中特色发展，以产教融合、校企合作为重点，大力发展职业教育。加强公共文化服务体系建设，实现文化惠民与群众文化需求有效对接，加快实施抗美援朝纪念馆改扩建工程。弘扬辽宁精神，广泛开展以“学习雷锋、奉献他人、提升自己”为主题的社会志愿服务活动，提高市民文明素质和城市文明程度。积极稳妥推进卫生体制改革，全面实施基本药物制度，抓好村镇卫生体系建设，落实计划生育政策。加强体育场馆等基础设施建设，做好省十二运承办及各类赛事组织工作，继续增加群众休闲运动场所。

　　创新社会治理。深化反渗透、反颠覆、反窃密斗争，严厉打击侵财、涉毒、黑恶和涉及民生等领域犯罪。整合现有电子监控资源，拓宽为民服务渠道。强化社会管理综合治理措施，做好社区矫正工作。加强民族团结，落实宗教政策。积极开展社会矛盾纠纷化解，强化信访责任，及时就地解决群众信访诉求。加强全民国防教育，深化国防动员工作，优化民兵预备役建设，继续争创双拥模范城，加大管边控边力度，确保边境地区和谐稳定。加快形成科学合理的食品药品安全体系，有效规范市场秩序，保持物价总体平稳。加大扶持力度，加快社会养老服务业发展。完成城市社区居委会换届选举任务。

　　狠抓安全生产。有效落实企业主体责任，加快整改市级重大安全隐患，严格事故查处和责任追究，加强安全培训教育，进一步提升应急救援能力，有效防范较大事故，杜绝重大以上事故的发生。

　　(九)加快建设为民务实清廉政府

　　让政府工作更加务实诚信。树立做官必须做事理念，把每一件事能否落实作为衡量领导干部工作的标准。注重可持续发展，尊重丹东实际，不搞劳民伤财的形象工程和脱离实际的政绩工程，让丹东的发展步伐更加踏实和坚定。坚持对丹东百姓负责，对未来负责，做必务实，行必长远，不透支资源、不污染环境，不给后人留负担，让丹东的青山绿水成为永续的财富。向群众承诺的事必须制定切实可行的实施方案，并及时公布进展情况，把好事办好，办得让群众顺心、满意。

　　让政府作风转变更加明显有效。严格执行中央“八项规定”及省委、市委关于改进作风的各项规定，把群众路线教育活动的落脚点放在真正解决问题、让老百姓得实惠上，群众反感的事坚决不做，群众不满意的现象坚决整改。做好公车、办公用房等方面的改革和清理，严格执行公务接待标准，促进“三公”经费大幅压缩。政府各类文件多用大众化语言，让群众看得明白、听得明白、算得明白。不开无实际效果的会，不开冗长拖沓的会。狠抓软环境建设，把政府部门和窗口单位提高服务质量作为今年转变作风的重点，对不耐心解释、政策把握不准的要抓好教育和培训，对故意刁难、推诿群众甚至利用手中权力索要好处的要严厉查处。

　　让政府机构更加便民高效。按照国家、省统一部署和要求，正确、准确、有序、协调推进改革，坚定不移地落实好改革各项措施，积极稳妥地完成改革工作任务。稳步向城区下放管理权，做好权力衔接，提高服务效能，使城区补齐短板、激发活力。抓好机构改革，完成工商局、质监局管理体制调整，以及食品药品监管局、卫生计生部门组建等改革任务。严格控制机构编制，财政供养人员总量只减不增。加快事业单位分类改革，扩大政府购买服务范围。深化行政审批制度改革，建立和完善市、县(市)区、乡镇公共行政服务体系，推行集中审批、阳光操作、高效便捷的审批方式。

　　让政府做的事更加公正透明。积极主动地接受人大法律监督和政协民主监督，虚心听取各方意见、建议。推进政务公开，政府重大事项决策过程让群众参与，执行过程让群众监督，办理结果让群众评判。领导干部多深入基层、深入群众，看看还有哪些工作没做好、没落实，听听老百姓还有哪些意见和要求，对市长信箱、民心网及市民投诉热线等渠道收到的群众诉求做到有问必答。强化监察工作，认真落实党风廉政责任制，深入推进“五大系统”建设，实施“推权进笼”工程，对重大决策部署执行情况进行跟踪审计、问效、问责，对审批事项、政府采购等重要环节加大监管和案件查处力度。强化依法治理和依法行政，有效规范行政执法行为。

　　各位代表，加快发展的使命激励着我们，深化改革的重任考验着我们，人民过上幸福生活的期待鞭策着我们，让我们在党的十八届三中全会精神指引下，在市委的正确领导下，在市人大、市政协的监督、支持下，进一步开拓进取，攻坚克难，奋力前行，不断推进大气秀美新丹东建设，为率先全面建成小康社会而付出更大的努力!
